# Multidisciplinary estimates of connectivity and population structure suggest the use of multiple units for the conservation and management of meagre, *Argyrosomus regius*

**DOI:** 10.1038/s41598-023-50869-9

**Published:** 2024-01-09

**Authors:** D. Abecasis, R. Ogden, A. C. Winkler, M. Gandra, B. Khallahi, M. Diallo, R. Cabrera-Castro, Y. Weiller, K. Erzini, P. Afonso, J. Assis

**Affiliations:** 1 CCMAR, Centre of Marine Sciences, University of Algarve, 8005-139 Faro, Portugal; 2 Royal (Dick) School of Veterinary Studies and the Roslin Institute, University of Edinburgh, Easter Bush Campus, Midlothian, EH25 9RG UK; 3 Institut Mauritanien de Recherches Océanographiques et des Pêches (IMROP), BP 22, Nouadhibou, Cansado Mauritania; 4 Conservation and Research of West African Aquatic Mammals (COREWAM), Dakar, Senegal; 5 Departamento de Biología, Facultad de Ciencias del Mar y Ambientales, Universidad de Cádiz. Campus de Excelencia Internacional del Mar (CEIMAR), Avda. República Saharaui, s/n, Puerto Real, 11510 Cádiz, Spain; 6 Instituto Universitario de Investigación Marina (INMAR), Campus de Excelencia Internacional del Mar (CEIMAR), Avda. República Saharaui, S/N, Puerto Real, 11510 Cádiz, Spain; 7 Parc naturel marin de l’estuaire de La Gironde et de la mer des Pertuis, OFB, 17320 Marennes, France; 8 Ocean Sciences Institute (Okeanos), University of the Azores, 9901-862 Horta, Portugal; 9 Institute of Marine Research (IMAR), 9901-862 Horta, Portugal; 10 Faculty of Bioscience and Aquaculture, Nord Universitet, Bodø, Norway

**Keywords:** Marine biology, Conservation biology, Animal migration

## Abstract

Information on population structure and connectivity of targeted species is key for proper implementation of spatial conservation measures. We used a combination of genomics, biophysical modelling, and biotelemetry to infer the population structure and connectivity of Atlantic meagre, an important fisheries resource throughout its distribution. Genetic samples from previously identified Atlantic spawning locations (Gironde, Tejo, Guadalquivir, Banc d’Arguin) and two additional regions (Algarve and Senegal) were analysed using genome-wide SNP-genotyping and mitochondrial DNA analyses. Biophysical models were conducted to investigate larval dispersal and connectivity from the known Atlantic spawning locations. Additionally, thirteen fish were double-tagged with biotelemetry transmitters off the Algarve (Portugal) to assess movement patterns and connectivity of adult individuals. This multidisciplinary approach provided a robust overview of meagre population structure and connectivity in the Atlantic. Nuclear SNP-genotyping showed a clear differentiation between the European and African populations, with significant isolation of the few known Atlantic spawning sites. The limited level of connectivity between these subpopulations is potentially driven by adults, capable of wide-ranging movements and connecting sites 500 km apart, as evidenced by tagging studies, whilst larval dispersal inferred by modelling is much more limited (average of 52 km; 95% of connectivity events up to 174 km). Our results show sufficient evidence of population structure, particularly between Africa and Europe but also within Europe, for the meagre to be managed as separate stocks. Additionally, considering the low degree of larvae connectivity, the implementation of marine protected areas in key spawning sites could be crucial towards species sustainability.

## Introduction

Intraspecific biodiversity (i.e., genetic diversity), is one of the three main pillars of biodiversity and plays an important role in species resilience, adaptation, and evolution^1^. Higher levels of genetic diversity correlate with the ability of populations to respond to environmental changes, such as those caused by ongoing climate change^2^ and with external add-on pressures (e.g., overfishing), thus increasing the chances of long-term species sustainability^3,4^. Inadequate management of exploited fish species can lead to over-harvesting, potentially resulting in population bottlenecks and reduced levels of genetic diversity^4,5^. Understanding extant population genetic structure and connectivity patterns is therefore crucial for the sustainable management of exploited species, and the implementation of effective conservation measures such as marine protected areas (MPAs)^6–8^.

Knowledge of how populations are geographically distributed can help determine the spatial extent of management units or the design of monitoring programs^9^. In marine systems, commercial fish stocks are closely associated with biological populations, and understanding their connectivity patterns has been recognized as an important aspect of effective MPA networks, to promote biodiversity, population resilience and persistence^10–12^. Investigating the spatial structure of biological populations requires data on a species’ life history, including larval and juvenile dispersal, adult migration, and genetic reproduction. For example, populations with distinct genetic structures might require separate management strategies. If two populations are not interbreeding due to a natural barrier conserving one might not safeguard the other.

In most marine fish, connectivity between regions occurs primarily during their larval phase through passive dispersal facilitated by oceanographic currents^13–15^, and this can be inferred with biophysical modelling e.g.,^13^ across all species ranges and regions^16^. In certain species, the migration of adult individuals can also play an important role in population structure through a mechanism known as adult mediated population connectivity^17^, which can result in greater genetic connectivity than through larval mediated processes in cases where adult migration occurs over larger distances. Methods to study lifetime individual movement such as biotelemetry and otolith microchemistry have advanced considerably over recent years helping to provide a clearer picture of population structure and connectivity^18^.

Genetic structuring within populations provides a window into the historical and current interactions among populations, influenced by both natural barriers and anthropogenic disturbances. Marine fish species have traditionally been considered to exhibit low population genetic structure, due to their high dispersal capabilities and the general lack of dispersal barriers^19,20^. Nevertheless, fish population distributions do have limits and mechanisms such as restricted dispersal or natal philopatry, which lead to repeated use of the same spawning grounds across generations, can increase population differentiation by reducing effective gene flow^9^. Furthermore, developments in molecular methods and the increasing number of DNA markers available for population genetic analyses have allowed employing evermore powerful approaches for the characterisation of within-species variation^21^, revealing increasing levels of genetic spatial variation in the ocean^13^. Information on population genetic structure is therefore now considered central to the adequate implementation of conservation and fisheries management efforts^10,14,22,23^. A deeper understanding of the genetic structure has profound implications for conservation strategies, ensuring they are evidence-based and tailored to the specific needs of species^5,24^. Differentiated metapopulations, characterized by distinct subpopulations, offer several advantages for resilience. Firstly, they often represent a broad range of genetic adaptations, reflecting the varied environments or niches that each subpopulation occupies. This breadth of genetic variation might be crucial for adaptability in the face of changing environments, overfishing or catastrophic events^24^. Yet, these metapopulations can also be vulnerable. Small, isolated populations are more susceptible to local extinction events due to stochastic factors. Reduced gene flow between them can also lead to inbreeding depression, reducing their overall fitness^25^. In contrast, a single large panmictic population, marked by widespread gene flow and genetic homogeneity, benefits from the genetic diversity arising from a larger population size^24^. Such diversity can be a buffer against a multitude of threats, ensuring that at least a fraction of the population has the genetic makeup to withstand challenges. Yet, the panmictic structure isn't without its challenges. Being a singular large unit, it might be more vulnerable to wide-ranging threats that affect the entire population. Conservation efforts for such structures might need to focus on broad-scale protections, ensuring the preservation of habitats and mitigating large-scale threats^24^.

Despite the wide array of physical, chemical, and biological approaches to investigating population biology, for most marine species their spatial patterns and mechanisms underlying population structure, particularly the extent of larval and adult connectivity, remain poorly understood. This is the case for meagre, *Argyrosomus regius*.

Meagre is one of the largest coastal bony fishes in the Eastern Atlantic, reaching over 180 cm in length and more than 50 kg in weight^26,27^. Meagre is distributed from the Bay of Biscay to Senegal, and across the Mediterranean Sea and the Black Sea^28^. Local extinction and decreases in the abundance of this species have been reported^29,30^, highlighting the need for data supporting well informed management and conservation strategies. To date, and despite its ecological and economic importance, biological and ecological information on meagre is still scarce, particularly regarding its population structure and connectivity. Current knowledge on the species’ population structure and migratory behaviour is based on indirect evidence from fisheries landings^27,31^, otolith geochemistry^32^, and one genetic study^28^. In summary, meagre forms large spawning aggregations during the spring and summer months in, according to the literature, six main sites: the Gironde (France), Tejo (Portugal) and Guadalquivir (Spain) estuaries, and the Banc d’Arguin (Mauritania) in the eastern Atlantic, and the Nile (Egypt) and the Menderes (Turkey) deltas in the Mediterranean^28^. After spawning, adults migrate to offshore areas where they spend autumn and winter months whilst juveniles stay in near-shore areas close to spawning grounds during their first two to four years of life^27,32–34^. The observed high genetic differentiation between populations of the Atlantic and the Mediterranean, suggests limited connectivity between these regions as also marked by an apparent genetic barrier between the west and south coasts of Portugal^28^.

The aim of this study was to examine population structure in meagre along the Atlantic coast using a multi-disciplinary approach to investigate genetic variation, larval dispersal, and adult migration. We used mitochondrial DNA sequencing analysis and nuclear DNA genotyping-by-sequencing to investigate levels of genetic differentiation and gene flow from France south to Senegal. Biophysical modelling was used to assess the potential of larval dispersal from the known spawning sites in the Atlantic (Gironde, Tejo, and Guadalquivir estuaries, and Banc d’Arguin). Biotelemetry methods, acoustic and satellite telemetry, were applied to investigate the movement patterns of adult meagre individuals along the Southern Iberian coast. Lastly, these three components were combined to help unravel the population structure and connectivity of meagre in the Atlantic, providing relevant information for the sustainable management of this important species both through fisheries management and area-based conservation measures such as MPAs.

## Materials and methods

### Genomics

**Figure 1:**
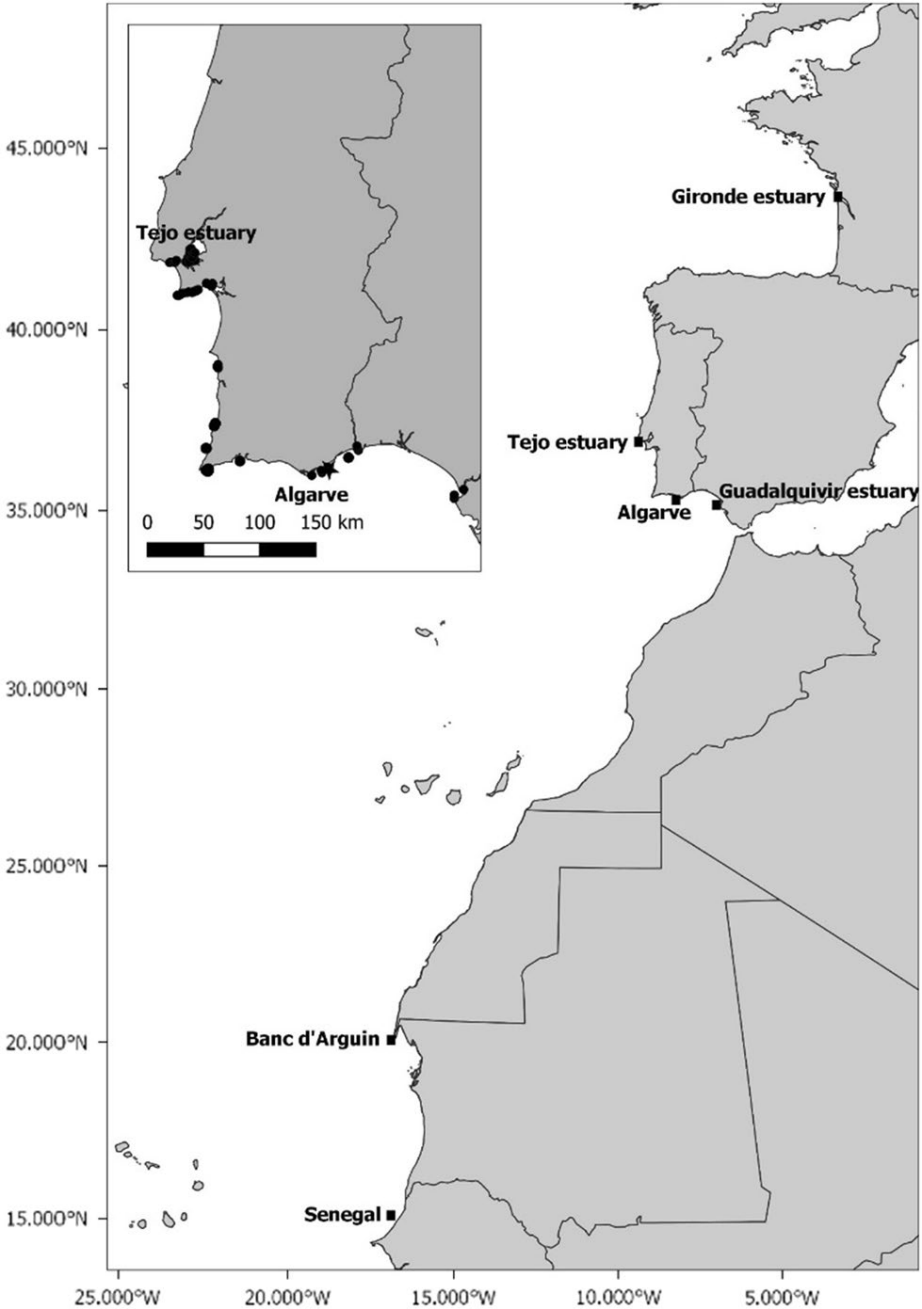
Map showing the location of *Argyrosomus regius* sampling sites (filled square). The inset shows the tagging location (star) and the location of the acoustic receivers (filled circle).

A total of 154 tissue samples of meagre were collected from the species’ known spawning sites in the Atlantic (Gironde, Tejo, Guadalquivir, and Banc d’Arguin) and two additional sites, Algarve and Senegal (Fig. 1). Samples were kept in 2 ml tubes containing ethanol and stored at – 80 °C until DNA extraction. All samples were subjected to DNA extraction using the Qiagen DNeasy Blood and Tissue extraction kit, following the manufacturer’s instructions. Samples were eluted into 100 µl of AE buffer and subsequently quantified using a Thermofisher Qubit fluorometer. Because DNA quantification values varied significantly among samples, with many samples returning relatively low DNA yields (< 20 ng/µl), we concentrated weaker samples using vacuum centrifugation and diluted the strongest samples using an additional elution buffer. Following normalisation, all samples were re-quantified and a subset of 94 samples was selected for downstream nuclear DNA analysis.

#### Mitochondrial DNA analysis

Mitochondrial control region sequencing was performed on 86 samples using a novel set of PCR primers designed for this project (Forward: Meag_CR_F 5′-TCTTTCTAGGGGGTCACTGG-3′; Reverse: Meag_CR_R 5′-CATCTTAACATCTTCAGTGTTATGCT-3′), which amplify around 330 base pairs of control region sequence previously observed to vary in a congeneric species (Henriques et al. 2015). PCR thermocycling was performed in 20 µl reaction volumes (10 μl 2× Dreamstart Mastermix (Thermofisher); 1 ml each primer (10 mM); 6 ml water; 2 ml DNA template; 35 cycles: 94 °C (30 s)–55 °C (30 s)–70 °C (60 s)) with the resulting PCR product cleaned with Exo-SAP (New England Biolabs) prior to bi-directional Sanger sequencing on an ABI3730 genetic analyser. Raw mtDNA CR sequence data were edited using Geneious v10.0.2 (Biomatters Inc.) and submitted to GenBank (Acc. No.s ZZZZZZ). Sequences were analysed to identify haplotype diversity and distribution using a combination of sequence diversity statistics, phylogenetic reconstruction, network analysis and mapping approaches. Sample sequences were aligned to one another, and haplotype diversity statistics were calculated using the software package Contrib^35^. The combined sequence alignment of 86 samples plus outgroup was subject to phylogenetic reconstruction performed in Geneious, using default parameters for alignment and the construction of Neighbour-Joining trees (Outgroup = *Argyrosomus coronus*, GenBank Acc. No. JX191938). Network analysis was performed in the software PopArt^36^ using the Median-Joining Network algorithm^37^ to examine haplotype relatedness and distribution across the six sampled localities. Pairwise F_ST_ was calculated among localities in Arlequin^38^.

#### Nuclear DNA analysis

A total of 94 samples and two replicate controls were submitted for DNA sequencing at Diversity Arrays Technology (Canberra, Australia) using the DArT sequence protocol, an approach whereby nuclear single nucleotide polymorphism (SNP) DNA markers are simultaneously discovered and characterised across an entire set of DNA samples^39^. The dataset generated by DArT sequence analysis was analysed using a bioinformatic pipeline. A single genotype dataset was created for population genetic analysis by applying a series of filters to remove failed samples and SNP markers of questionable quality or low information content (see supplementary material S1 for details).

The resulting genotype dataset was analysed to examine levels of population genetic structure by means of STRUCTURE analysis^40^ to infer the putative number of populations (K) based on microsatellite data. Three independent runs per K (ranging 1–6) were performed using 500,000 MCMC iterations after a burn-in of 200,000 replicates, using an admixture model with correlated allele frequencies without prior sampling information. The most likely number of genetic clusters, K, was assessed using the software STRUCTURE HARVESTER^41^. Ordination analysis with and without information of the geographic sample origin, Discriminant Analysis of Principal Components (DAPC) and Principal Coordinates Analysis (PCoA) respectively, were performed. The DAPC^42^ was conducted using the adegenet package^43^ for the R software version 3.5.2.^44^ and the PCoA analysis was performed in Genalex 6.5^45^.

To determine the level of genetic differentiation between pairs of populations, F-statistics^46^ were calculated using ARLEQUIN v 3.5.2^47^. F_ST_ values were obtained from variance in SNP allele frequencies. Finally, isolation by distance (IBD) patterns based on SNP genotypes were examined to determine association between matrices of pairwise codominant genotypic and geographic (Euclidean) distances by performing a simple Mantel test (1000 permutations) computed using Genalex 6.5^45^. Where population structure was apparent through observed reductions in gene flow between two localities, additional outlier analyses were performed using the software Bayescan^48^, which assesses the distribution of pairwise F_ST_ values across all individual SNPs in the dataset to identify those markers that significantly deviate from expectations, identifying markers that show particularly high or low F_ST_ values. Such markers may be associated with areas of the genome under selection.

Standard indices of genetic diversity were also reported, namely number of alleles (Na), rarefied allelic richness (Ar), observed (Ho) and expected (He) heterozygosity across all SNP loci and regions, using Genalex 6.5^45^. Conformation to Hardy–Weinberg equilibrium (HWE) and linkage disequilibrium (LD) were tested by performing exact tests in GENEPOP, version 4.2^49^ with default settings for Markov chain parameters.

### Larval biophysical modelling

Biophysical modelling was developed to estimate larval dispersal and potential connectivity between the known Atlantic spawning locations, based on a widely implemented framework e.g.,^13,16^, with openly available protocol and source code^50^. This fed on the Hybrid Coordinate Ocean Model (HYCOM version 2.2; for additional information please refer to Assis 2022), which delivers daily data on the direction and intensity of ocean currents with a spatial resolution (0.08°) allowing the capture of the broad patterns of population connectivity^51,52^ and structure across marine taxa e.g.,^13,53–55^.

The biophysical model estimated dispersal through individual particles representing larvae advected by ocean currents. The particles were released on a daily basis during the known spawning season, from sites with approx. 3 km side (here defined as source sites) generated within the limits of the known spawning locations. A high-resolution polygon defined the landmasses of the study region^56^. The position of each individual particle was determined every hour of simulation by using bilinear interpolation over the eastward and northward components of the HYCOM velocity fields e.g.,^16^. Particles drifted for a period of 30 days until eventually ended up on landmass, a coastal area including known spawning locations or got lost in the open ocean. To account for interannual variability, simulations run for a 10-year period (2008–2017). The duration of the spawning season for each location was obtained from the literature and ranged between June and mid-July for the Gironde and between May and August for Banc d’Arguin^27,57–59^.

At the end of the simulation, we estimated the average and the maximum dispersal distances for each spawning location. The trajectories of all particles were aggregated into pairwise probability matrices of oceanographic connectivity between spawning locations, by determining the proportion of particles released from a given spawning location that reached an alternative spawning location.

### Biotelemetry

All tagging procedures were conducted following protocols approved by the Centre of Marine Sciences (CCMAR) Animal Welfare Commission. Additionally, the tagging work conducted for this study was approved by the General Directorate of Food and Veterinary (DGAV), including its ethical committee, under the permit 2018/08/29 015730. Fish were captured under permits 561/018/CAPT and 142/2019/CAPT by the Institute for the Conservation of Nature and Forests (ICNF). Fish were tagged in accordance with guidelines and regulations on animal experiments as stated in the corresponding permits and all work was conducted according with the ARRIVE guidelines.

A total of 13 adult meagre were double tagged with acoustic and pop-up satellite archival (PSAT) transmitters. We focused on tagging adult specimens to study post-recruitment dispersal, as juveniles are believed to inhabit nearshore zones adjacent to spawning grounds until reaching an age of two to four years^27^. Fish were passively captured in a tuna trap located offshore Olhão (Algarve, Portugal) in September 2018 (n = 6), July 2019 (n = 2) and September 2019 (n = 5). An acoustic transmitter (Vemco V16-5x) with an average signal emission of 90 s and an expected lifetime of 1292 days was surgically implanted into the abdominal cavity. For this purpose, fish were placed inverted in a soft stretcher with running water passing through its gills and mouth, and an incision made in the ventral region. After the insertion of the acoustic transmitter, the incision was closed using absorbable sutures (BBraun, Novosyn). Afterwards, a PSAT (Wildlife Computers Minipat) was attached externally, below the dorsal fin, using a small titanium anchor for details see^34^. Fish were measured (TL) and tagged with an external dart tag before being released in the capture location. No anaesthesia was used for these procedures.

Detections of acoustically tagged meagre were obtained through an array of acoustic receivers placed throughout the coastal area of southern Portugal (Fig. 1). The PSATs recorded light level, depth, and water temperature every 3 or 5 s and summarised these data every 6 h for transmission. Tag detachment was programmed to take place after 120, 180 or 300 days after tagging. Once the tags reached the surface data was transmitted via the Argos satellite system. Position estimates, based on the data collected by the PSAT on light-level, SST and depth, were obtained using the HMMoce package^60^. Whenever acoustic detections occurred during the PSAT sampling period they were included as known locations to improve the model accuracy (see Gandra et al. submitted for further details).

## Results

### Genomics

#### Mitochondrial DNA

**Figure 2:**
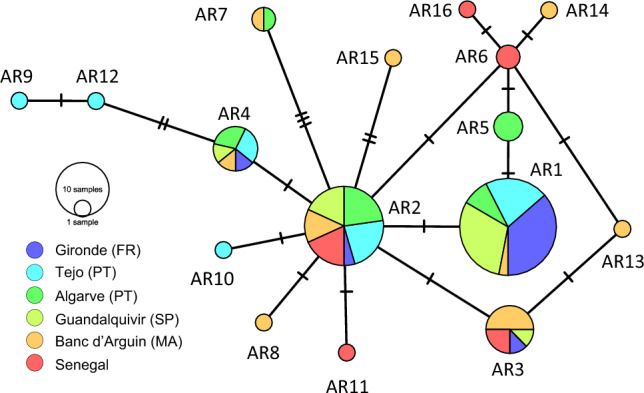
Relationships among the sixteen *Argyrosomus regius* mtDNA CR haplotypes displayed in a median-joining haplotype network. Each node represents a haplotype (AR1–AR16), node size proportional to sample number. Bars across connections represent the number of sequence mutations between haplotypes.

**Figure 3:**
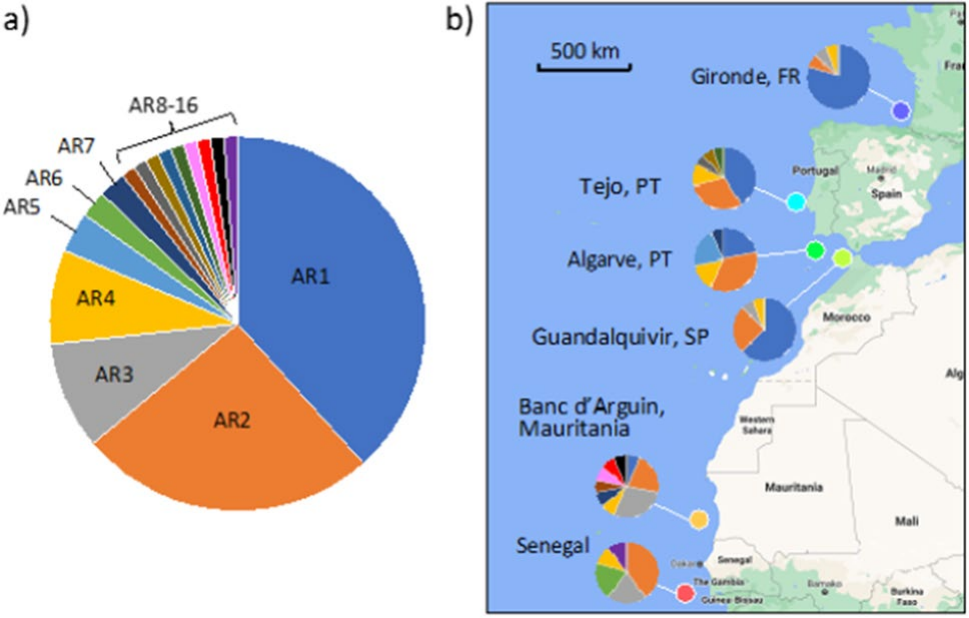
(**a**) Relative proportions of the sixteen *Argyrosomus regius* haplotypes observed across the 86 samples sequenced, showing the dominance of several common haplotypes within the data set. (**b**) Map showing the distribution of mtDNA CR haplotypes diversity observed across six localities along the coast.

A total of 86 DNA sequences were resolved across the six sampling locations, revealing a high level of mitochondrial genetic diversity with sixteen mtDNA control region haplotypes (hereafter AR1 to AR16). Shallow genetic diversity differences were found, with the northernmost locality (Gironde) having the least diversity and the southernmost localities (Banc d’Arguin and Senegal) the most (Table S3). The haplotype network displayed a complex set of relationships among the haplotypes and localities; no clear phylogeographic pattern was evident, with multiple localities displaying the same common haplotypes, AR1, AR2, AR3 and AR4, and the sixteen haplotypes showing very similar levels of sequence divergence from one another (Figs. 2, 3). The distribution of haplotypes shows some variation in the presence and proportion of individual haplotypes observed at each locality, for example, haplotype AR1 is at a particularly high frequency off the European locations but is at low frequency in Banc d’Arguin and absent from Senegal. However, haplotype AR2 is observed at all six localities and there is no striking geographic structure evident from the distribution of haplotypes by locality (Fig. 2). Pairwise F_ST_ data reveal significant differentiation at 9 of 15 locality pairs; however, there is no consistent geographic pattern to this genetic structuring (Fig. S3). For example, while Tejo and the Algarve showed significant differentiation from Gironde, Guadalquivir, which is more distant, did not. No significant pairwise F_ST_ values were observed among certain neighbouring localities (Tejo-Algarve-Guadalquivir and Banc d’Arguin-Senegal).

#### Nuclear DNA SNP

Initial DArTseq processing generated a total of 13,457 sequences containing SNP markers with genotype data across the majority of the 94 samples. Sequential filtering reduced this number to 1,534 SNPs with a call rate above 95%, Minor Allele Frequency above 5% and mean reproducibility of 99% (Supplementary Data S1, Table S1). One sample from the Algarve and six from Senegal were excluded from further analyses due to poor genotyping results. The final dataset of 1,534 SNPs and 87 individual samples (Gironde (n = 12); Tejo (n = 23); Algarve (n = 12), Guadalquivir (n = 16); Banc d’Arguin, (n = 17), and Senegal (n = 7)) was subject to population genetic analyses to investigate local diversity and putative geographic structure.

**Figure 4:**
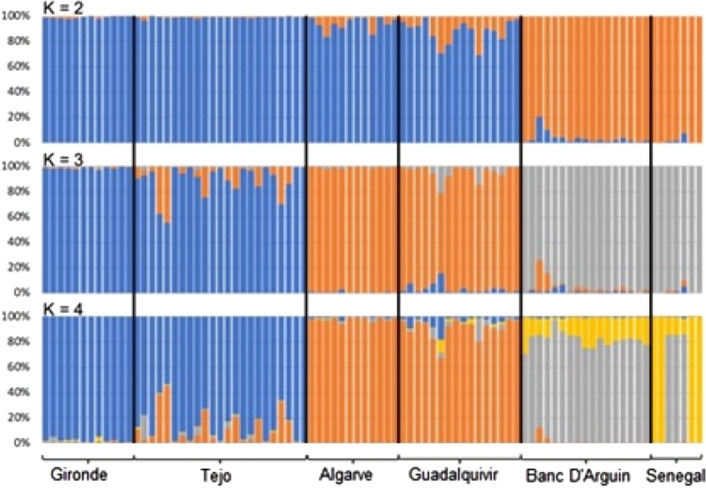
Results of the STRUCTURE analysis showing the distribution of individual genetic variation among different putative genetic clusters. Individual genotypes are represented by vertical bars along the x-axis, with the proportion of membership to K genetic clusters indicated by the different colours on the y-axis. Individuals are grouped by localities along the x-axis corresponding to Gironde, Tejo, Algarve, Guadalquivir, Banc d’Arguin and Senegal.

Test for deviations from Hardy–Weinberg Equilibrium revealed 186 loci showing significant (P < 0.01) heterozygote deficiency in single populations, sixteen loci showed deviation in two populations and two loci showed deviation in three populations, from a total of 9204 tests. None of these were significant after Bonferroni correction for multiple tests. Tests for linkage disequilibrium showed no significant association between genotypes across SNP loci across all populations. No loci were removed from the analysis based on these results. The ΔK criterion^61^ of the STRUCTURE analysis showed the existence of two main populations in the Atlantic: the European and the African populations (K = 2; Fig. 4; Supplementary Data S2; Table S2; Figs. S1, S2).

**Figure 5:**
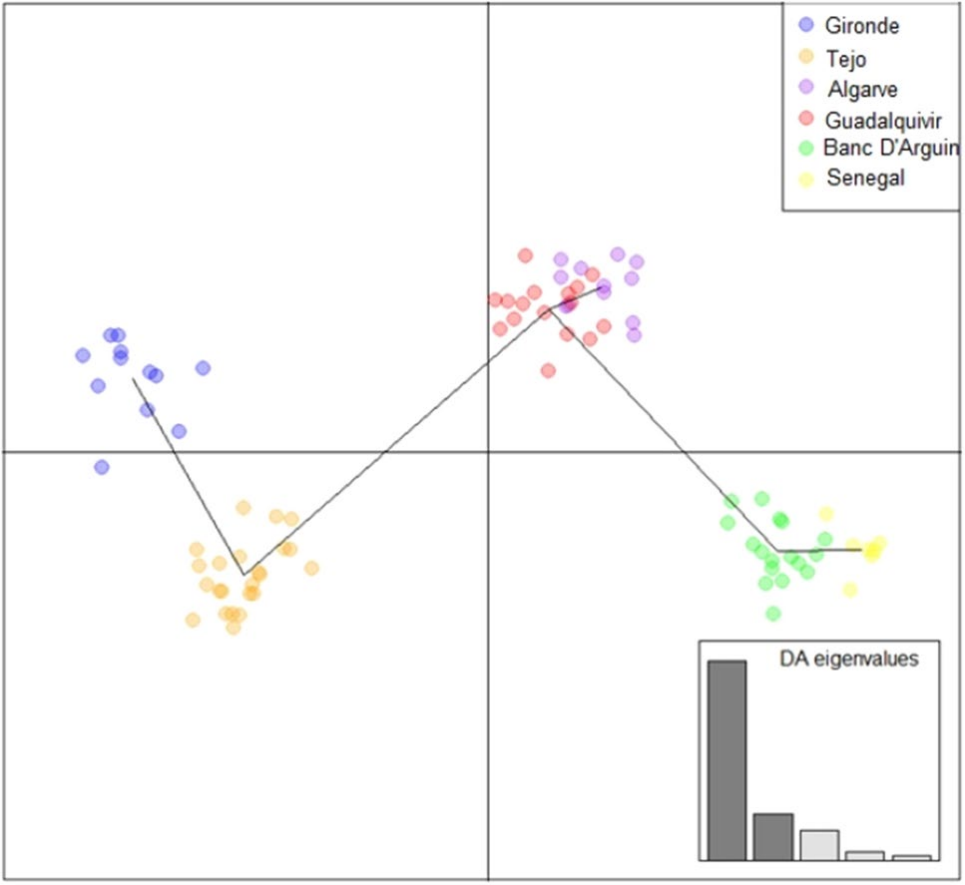
DAPC plot showing the distribution of samples associated with each sampling locality plotted along the first two principal components.

The DAPC and PCoA supported the existence of these two populations, and showed an additional higher level of genetic structure, subdividing the species in three (PCoA; Fig. S4) or four genetic clusters (DAPC; Fig. 5), also partially supported by STRUCTURE (K = 3 and K = 4; Fig. 4). When the population genetic analyses were informed by locality information (i.e., DAPC analysis), population substructure among localities becomes much more pronounced, with clear separation of samples from Gironde and Tejo.

**Figure 6:**
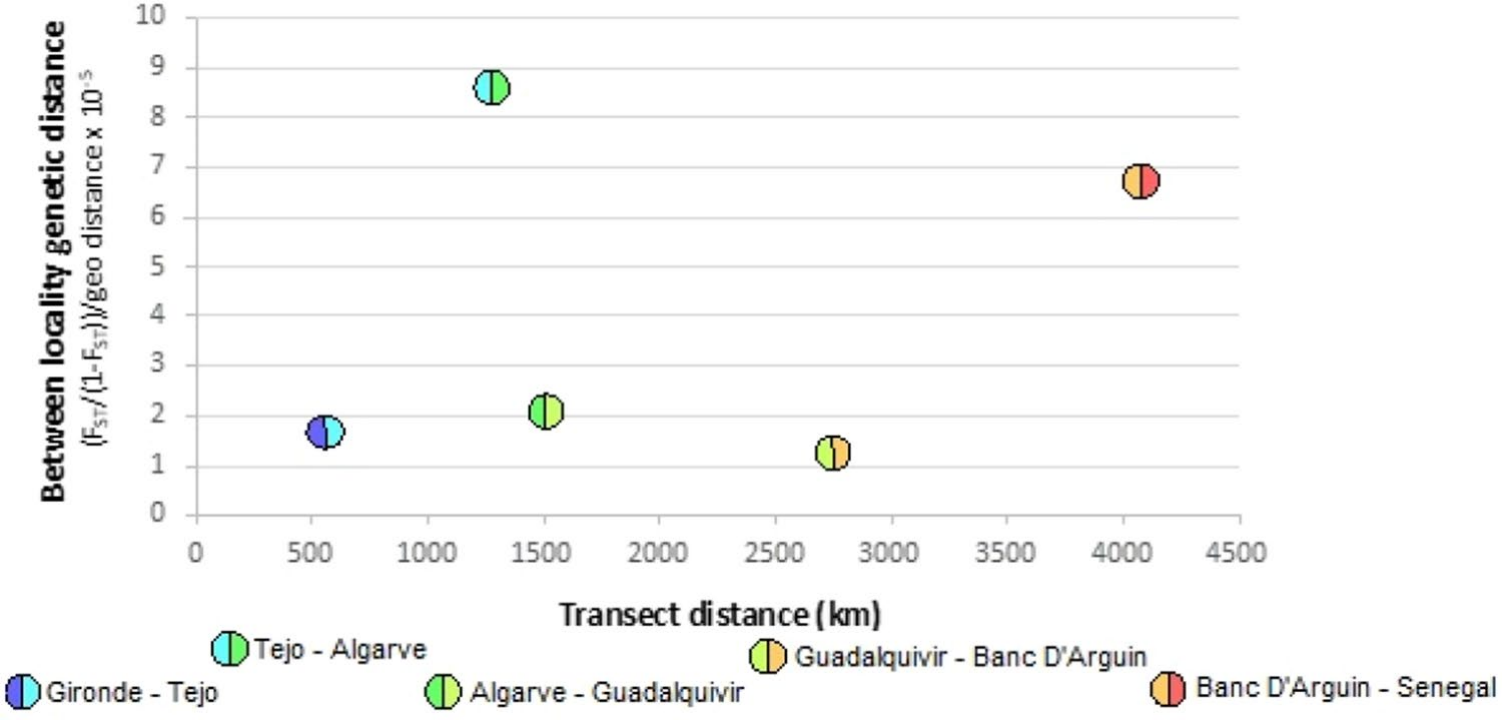
Standardised pairwise population genetic differentiation along a north–south transect matching the Atlantic coastline (left to right). Pairwise genetic distance (y-axis) is plotted against the geographic mid-point between localities (x-axis), showing reductions in gene flow (high F_ST_ values) relative to geographic distance.

Genetic differentiation levels among localities estimated with pairwise F_ST_ values were largely congruent with the genetic clustering analyses. All locality pairs displayed significant levels of population differentiation (significantly greater than zero), with higher values among the distant localities (e.g., Gironde and Senegal) and lower values among geographically proximate localities (e.g., Algarve and Guadalquivir) suggesting an isolation-by-distance pattern (Table S4). The inter-locality genetic divergence (measured as standardised pairwise F_ST_) revealed differences in potential gene flow between locality pairs along a north–south transect, with a substantial decrease in gene flow between Tejo and the Algarve and, to a lesser extent, between Banc d'Arguin and Senegal (Fig. 6).

BayeScan results showed a number of individual SNP markers displaying significantly elevated F_ST_ values; five SNPs with P < 0.01 and a further four with P < 0.05 (Fig. S5). These nine SNP markers were further investigated for significant association with protein coding regions using a series of BLAST analyses (BLASTn and BLASTx). BLASTn analysis returned matches between the outlier SNP sequences and existing sequence data from the congeneric *Argyrosomus japonicus* (‘Mulloway’; n = 8 SNPs) and *Larimichthys crocea* (‘yellow croaker’; n = 1 SNP) (Table S5). The results of BLASTx analysis, which searches nucleotide sequences against a protein database, found no significant matches for any of the nine outlier SNP DNA sequences. Removal of the nine outlier loci from the main dataset made no difference to the observed patterns of population genetic structure. Nuclear DNA SNP diversity estimates (heterozygosity and allelic richness) showed consistency among sampling localities, although Senegal displayed fewer polymorphic loci and mean number of alleles per SNP within the samples possibly due to the reduced sample size of this locality (Table S6).

#### Larval biophysical modelling

**Figure 7:**
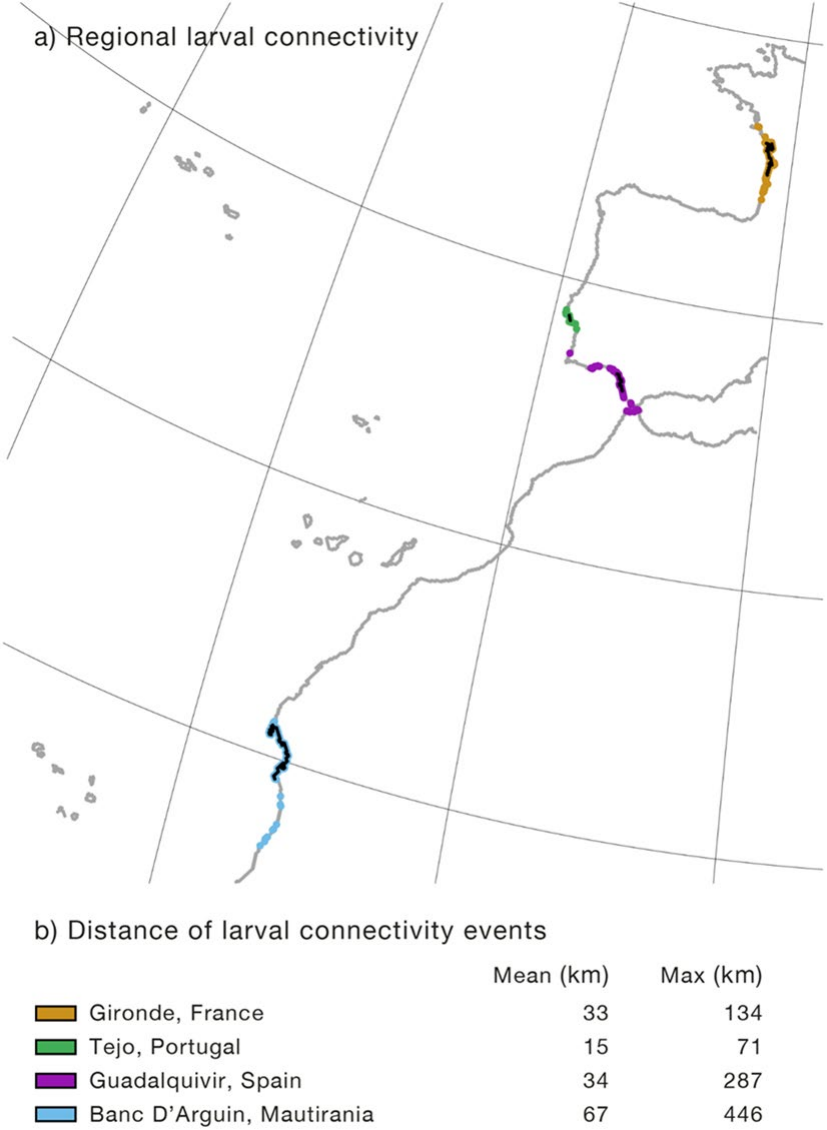
Panel (**a**) Meagre larval dispersal from each of the four known spawning sites in the Atlantic (Gironde—orange, Tejo—green, Guadalquivir—purple and Banc d’Arguin—blue) estimated by biophysical models. Darker areas represent larval source areas (spawning sites). Panel (**b**) Mean and maximum distances of *Argyrosomus regius* larval dispersal estimated with biophysical modelling for the main spawning locations in the Atlantic.

The biophysical model showed larval dispersal only at regional scales (dispersal events occurring at 51.83 ± 56.20 km, on average, and 95% of events up to 174 km; Fig. 7; Fig. S6), with no connectivity inferred between the main spawning locations of the Atlantic. Larval dispersal distances of the spawning locations ranged between 15 and 67 km with maximum distances of up to 446 km (Fig. 7).

#### Biotelemetry

Data from all but two PSAT tags was successfully retrieved either via satellite uplink or by physically recovering the tag, with most satellite tags reaching at least 90% of their predetermined deployment period (Table S7).

**Figure 8:**
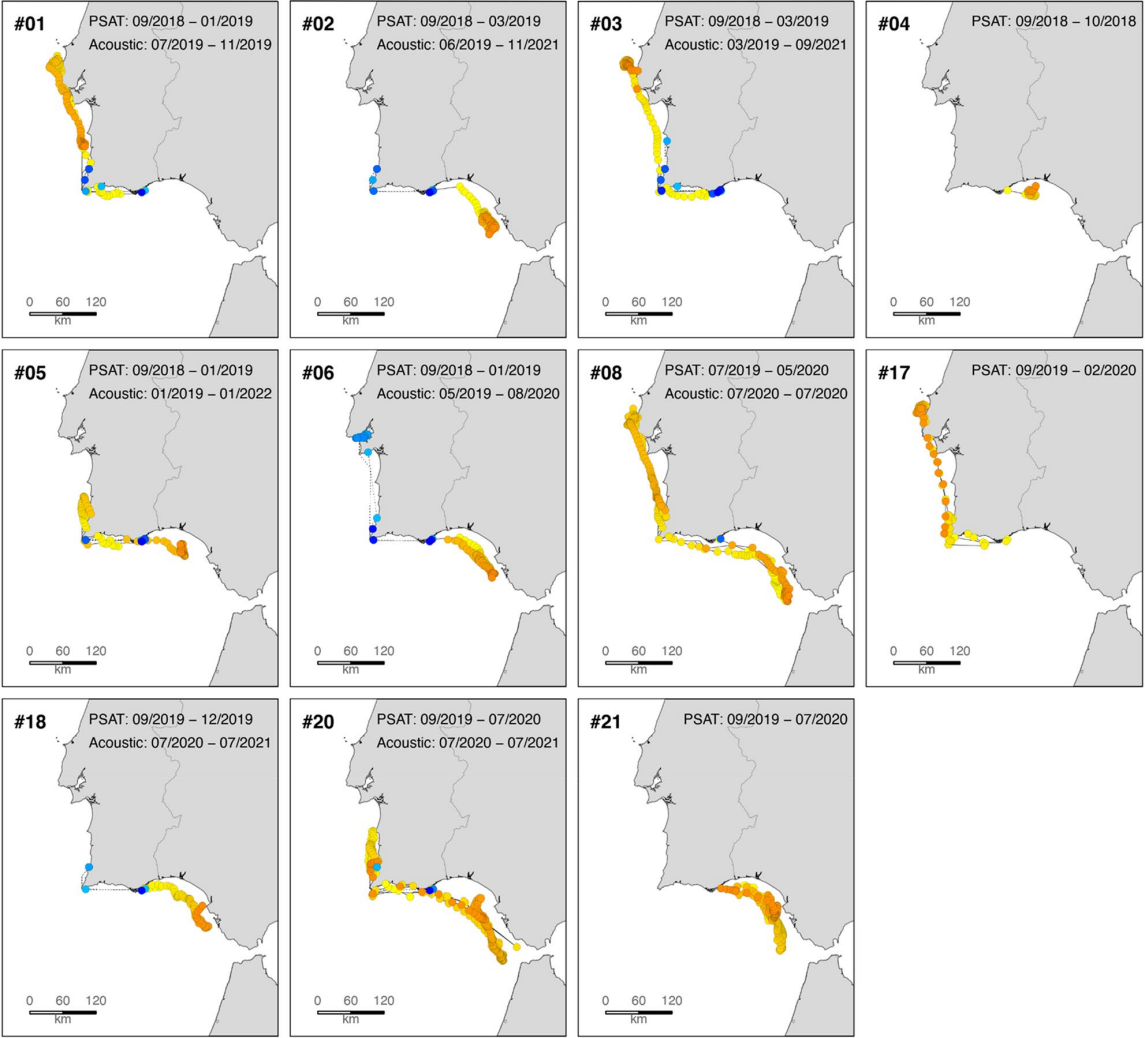
Movement patterns of *Argyrosomus regius* double tagged with acoustic and satellite transmitters off the Algarve coast. Location estimates derived from satellite data are shown from yellow (oldest) to orange (most recent), movement patterns between acoustic receivers shown from light blue (oldest) to dark blue (most recent). Two of the satellite transmitters never transmitted. Note: movements based on acoustic detections are only shown for the period after the satellite transmitter pop-up.

The results of the biotelemetric studies evidenced movements between the South and the West coasts of Portugal for 9 out of the 11 tagged individuals for which data was obtained (Fig. 8), including movements between two of the key spawning areas for this species, the Tejo and Guadalquivir estuaries. Tagged meagre were detected in locations over 500 km apart, covering distances up to 2000 km in a single year (Fig. 8 and Table S7). The use of PSAT and long-life acoustic transmitters allowed us to identify back and forth movements between the South and West coasts of Portugal with several individuals covering a total of more than 1000 km in 2 years.

## Discussion

To date, information on meagre population structure and connectivity was scarce and based on a single study using nuclear DNA microsatellite markers showing significant genetic differentiation between the Mediterranean and Eastern Atlantic, along with the presence of localised population structure in the Portuguese coast^28^. By combining DNA analyses with larval dispersal modelling and biotelemetry, as recommended for assessments of fish population structure and connectivity^14,62^, we show that the meagre’s Atlantic population presents significant genetic structure and low connectivity potential. The inferred levels of population differentiation are largely mediated by reduced larval dispersal with strong isolation of the few known spawning sites, despite larger potential for adult connectivity.

### Population structure and connectivity

The data from the mitochondrial control region revealed multiple haplotypes dispersed throughout the coastal locations, but inconsistent or patchy geographic structuring. The two African localities showed significant differentiation from most, but not all European localities. Similarly, Gironde was distinct from all but one locality (Guadalquivir), which disrupted a clear pattern of geographic differentiation. The network analysis found little to no geographic pattern in the genetic relationships among haplotypes, contrary to what may be predicted with significant population genetic structure.

The nuclear data showed much clearer differentiation between the European and African populations of the Atlantic, evident from both STRUCTURE and PCoA results, adding to the previously identified differentiation between the Atlantic and Mediterranean populations^28^. The fact that neither analysis uses locality information to support clustering highlights the strength of this differentiation in the dataset. When location data was taken into consideration (i.e., DAPC analysis), population substructure between sites became more obvious, with samples from Gironde, Tejo, Guadalquivir and Banc d’Arguin clearly separated. When compared to the prior study^24^, our F_ST_ results reveal a more pronounced differentiation among Atlantic populations, likely attributable to the higher resolution afforded by our methodology (1534 SNP vs 11 microsatellite markers). Our results highlight the importance of each of the known spawning locations, given their significant levels of genetic differentiation and the lack of broadscale connectivity. Accordingly, potential population declines in the face of environmental changes and/or other external pressures may result in drastic regional losses of genetic diversity, which in term may lead to losses of adaptive ability^63^.

The observed pattern of genetic structure along the Atlantic coast may be due to the long-term effects of isolation-by-distance (IBD), by which more distant population pairs show greater genetic variation due to a reduced opportunity for gene flow, relative to more proximate localities^64^. This analysis indicated a broad, significant, relationship between genetic and geographic distances, confirming that the greater the distances among localities the greater the level of genetic divergence. However, the inspection of the inter-locality genetic divergence, revealed differences in the level of gene flow between locality pairs along a north–south transect, with a marked reduction in gene flow between Tejo and the Algarve, and to a lesser extent between Banc d’Arguin and Senegal. Despite observing a small number of outlier SNP markers in the dataset, they did not appear to be driving these observed patterns. The overall levels of divergence observed are still quite low and would indicate that at least some migration of individual fish among localities is maintained, preventing higher levels of population structure from developing. While the observed pattern between Banc d’Arguin and Senegal is likely driven by the Senegalese sample outliers, for the Portuguese localities (Tejo and Algarve) it is indicative of a significant level of population structuring along the coastline, as also suggested by Haffray et al.^28^, which is supported by the lack of larval connectivity, as inferred by biophysical modelling. Given the observed movements of adult individuals between the West and South coasts of Portugal, the existing differentiation between these two populations could potentially be explained by the existence of natal philopatry, where individuals from different subpopulations share wintering grounds but reproduce in their natal estuaries, as observed in other sciaenid species^65,66^.

All sampling sites had similar nuclear genetic diversity levels, suggesting that either none of the examined populations are experiencing severe declines or that they are all under similar levels of pressure (e.g., fishing effort). Given that meagre are heavily targeted at all sampling sites particularly during spawning aggregations, and that large reductions in population size have been documented^29,30^, we hypothesise that fishing pressure is equivalent (and high) at all locations.

### Implications for management and conservation

Although considered as Least Concern by the International Union for Conservation of Nature^67^, meagre has faced local extinctions and decreases in abundance^29,30^. These highlight the need to put in place effective management and conservation measures to prevent further collapses. Based on the combined results of the mitochondrial DNA haplotype and nuclear DNA SNP datasets presented here, it can be concluded that there is evidence for population structuring in meagre along the East Atlantic coast. While mitochondrial DNA suggests recent population mixing, the observed structuring in nuclear DNA likely represents earlier historical events. Recombination in nuclear DNA, homogenizing allele frequencies, obscures signals of recent gene flow, providing insight into the complex population dynamics along the East Atlantic coast.

Our results suggest that meagre presents a strong dependence on just a few spawning locations where larval dispersal is low, and where adult individuals forming large aggregations are highly targeted^27,31^ putting in risk local stock viability in the long term^24,68^. The erosion of population structure via overharvesting of local populations or other local catastrophic events can lead to changes in population abundance and affect its resilience and recovery potential^24,62^. With such limited larval connectivity between known spawning locations, each spawning site effectively acts as a separate genetic reservoir. It is, therefore, imperative to provide adequate protection to the known spawning locations in order to keep genetic diversity and ensure species long term sustainability. The implementation of marine protected areas in the main spawning locations, or temporal closures during the spawning period, could contribute to long-term species viability by protecting the main larvae source areas^10^. This measure could be of extreme importance given that in these areas (i) adults form large aggregations where they can be easily targeted, (ii) larval dispersal occurs at regional scales only, and (iii) act as nursery grounds.

In contrast to larval behaviour, adult meagre exhibits wide-ranging movements, even connecting distant spawning locations. While this might suggest potential for genetic mixing, it is important to recognize that these movements do not necessarily equate to successful interbreeding or gene flow, as suggested by our results. Furthermore, wide-ranging movements can expose adults to a variety of threats (e.g., stationary fishing gears such as tuna traps) across larger geographic scales. Considering the extensive movement of adults, very large MPAs would be necessary to protect both breeding and feeding grounds^69^.

Our findings add nuance to the single large or several small (SLOSS) debate in conservation. For species exhibiting a metapopulation genetic structure, a “several small” conservation strategy might be suitable, preserving the unique genetic adaptations of each subpopulation. In contrast, species leaning towards a panmictic structure might benefit more from “single large” reserves, ensuring the protection of their broad genetic diversity. For meagre, the SLOSS debate leans toward “several small” reserves around each key spawning location, given the genetic differentiation and limited larval connectivity. The intriguing life history of meagre, with low dispersal larvae and far-traveling adults, presents unique conservation challenges. Addressing these effectively requires a multi-pronged strategy, blending site-specific protections with broader-scale measures. Our findings emphasize the intricate balance of localized protection and wider-scale considerations to ensure the resilience of this ecological and economically important species.

Regarding fisheries management, our results show that there is sufficient evidence for population differentiation, particularly between Africa and Europe, for the meagre to be managed as separate stocks at this scale. Within these regions, the patterns of population differentiation and connectivity among European localities also support division into separate management units between Gironde, Tejo and Algarve/Guadalquivir. Similarly, differentiation of Banc d’Arguin and Senegal may be justified for management purposes in terms of independent units of production, but their genetic differentiation is likely to be more related to geographic isolation than deeper evolutionary divergence. The inclusion of our results in stock assessments could contribute to improved fishery management plans and thus ensure the sustainability of this important resource^6,62,70^.

### Limitations and future work

Despite the importance of our results for the management and conservation of meagre, the study presents some limitations that should be acknowledged. Genetic sample sizes per locality were anticipated to be sufficient for the levels of diversity typically observed in marine fish, however given the number of mitochondrial DNA haplotypes observed here, more samples would be needed to ensure that all haplotypes present at each locality were sampled, and to generate accurate estimates of haplotype frequency. For example, haplotypes observed only once in the dataset were not informative in terms of population structure but may prove to be geographically restricted if the sample size were increased significantly. Moreover, the geographic distribution of the genetic sampling did not include Morocco leading to a large sampling gap between Spain and Mauritania, with this area suggested to harbour additional spawning sites^28^. The biophysical modelling approach, based on high-resolution ocean circulation data and specific pelagic larval duration and spawning locations, provided unique information regarding meagre larval dispersal from the known Atlantic spawning sites. Yet, additional drivers like active larval behaviour, competency or mortality that may also play an important role in larval dispersal^71–73^ were not considered due to lack of available information. Biotelemetric studies focused on a single tagging region and thus precluded us from inferring on adult dispersal movements in other regions. However, despite the reduced number of individuals tagged, the results provided clear evidence of adult connectivity between the South and West coasts of Portugal including movements between two spawning locations (Tejo and Guadalquivir), revealing that adult individuals can cover large distances (> 1000 km) and connect different spawning sites.

Future studies should focus on the putative existence of natal philopatry and on the impact of climate change on meagre distribution. Insights on the existence of natal philopatry, for instance via long term biotelemetric studies or otolith microchemistry, would help to better understand the effects of dispersal movements of adults towards population structure. Climate change can also have a role on meagre’s distribution and sustainability and predicting its impact can be crucial to implement adequate management measures. It is recognized that, in general, climate change will lead to reduced larval dispersal due to shorter pelagic larval durations^74,75^ which could mean a further decrease to the already short larval dispersal of meagre. Additionally, future climatic conditions could lead to significant range shifts toward the North^76^ rendering the current southern aggregations sites unsuitable for meagre and thus drastically reducing the number of spawning locations.

### Supplementary Information

Supplementary Information.

## Data Availability

The datasets used and/or analysed during the current study are available from the corresponding author on reasonable request.
