# Supplementary material for: Multidisciplinary estimates of connectivity and population structure suggest the use of multiple units for the conservation and management of meagre, Argyrosomus regius
Source: Sci Rep. 2024 Jan 9;14:873. doi: 10.1038/s41598-023-50869-9 (PMC10776566; doi:10.1038/s41598-023-50869-9)
Supplement: Supplementary file 1 — Supplementary Information. [file 41598_2023_50869_MOESM1_ESM.docx]

Multidisciplinary estimates of connectivity and population structure suggest the use of multiple units for the conservation and management of meagre, *Argyrosomus regius*

Abecasis, D.^a,*^, Ogden, R.^b^, Winkler, A.C. ^a^, Gandra, M. ^a^, Khallahi, B.^c^, Diallo, M.^d^, Cabrera-Castro, R.^e,f^, Weiller, Y.^g^, Erzini, K. ^a^, Afonso, P.^h,i^, Assis, J. ^a,j^

**Supplementary material**

**Supplementary Data 1: SNP filtering and genotype dataset production**

Following receipt of the processed raw data, a genotype dataset for population genetic analysis was created through the application of a series of filters to remove failed samples and SNP markers of questionable quality or low information content.

Filters applied in sequence:

Reproducibility Threshold = 90%. DArTseq™ runs 30% of the samples in replicate in independent libraries and sequencing runs, and the consistency of each locus is measured across these replicates (i.e., reproducibility). Loci that were not 90% reproducible were removed from the dataset.

SNP call rate Threshold = 95%. All SNPs present in <95% of samples were removed.

Failed samples Samples displaying high levels of bacterial contamination (and very low levels of target meagre DNA) were removed. This resulted in the removal of one sample from the Algarve and six from Senegal.

MAF Threshold = 1%. In a first phase of filtering, SNPs displaying a Minor Allele Frequency (MAF) below 1% were removed from the dataset. This equates to the removal of SNPs where the minor allele was only observed once, making the markers uninformative.

Threshold = 5%. In a second phase of filtering, SNPs displaying a Minor Allele Frequency (MAF) below 5% were removed from the dataset. This equates to the removal of SNPs where the minor allele was only observed eight times, minimizing the usefulness of the markers.

Table S1. Impact of SNP filtering on number of DNA markers retained:

Filtering step SNPs retained

Initial data 13,457

Reproducibility (90%) 13,410

Call rate (90%) 10,318

Call rate (95%) 4,196

MAF (1%) 2,690

MAF (5%) **1,534**

## **Supplementary Data 2: STRUCTURE Harvester output summary**

The results of STRUCTURE analysis of the meagre SNP data set and subsequent analysis of STRUCTURE output using the programme STRUCTURE HARVESTER ^1^ are summarised here.

Table S2. STRUCTURE run summary: Burn-in 200,000 Reps 500,000

# K Reps mean est. LnP(Data) stdev est. LnP(Data)

1 3 -124537.900000 1.819341

2 3 -123273.400000 11.363538

3 3 -122882.033333 29.097136

4 3 -122442.366667 191.023306

5 3 -122050.800000 42.565596

6 3 -122558.333333 245.920502

# File name Run # K Est. Ln P(data) Mean Ln (K) Var(Ln(lk)

200_500_nopop_run_7_f 7 1 -124536.8 -124158.2 757.1

200_500_nopop_run_13_f 13 1 -124540.0 -124158.2 763.6

200_500_nopop_run_1_f 1 1 -124536.9 -124157.9 757.9

200_500_nopop_run_14_f 14 2 -123280.6 -122400.2 1760.7

200_500_nopop_run_8_f 8 2 -123260.3 -122407.4 1705.8

200_500_nopop_run_2_f 2 2 -123279.3 -122404.1 1750.5

200_500_nopop_run_3_f 3 3 -122860.8 -121533.2 2655.2

200_500_nopop_run_15_f 15 3 -122915.2 -121534.3 2761.7

200_500_nopop_run_9_f 9 3 -122870.1 -121530.1 2680.1

200_500_nopop_run_4_f 4 4 -122335.8 -120615.7 3440.3

200_500_nopop_run_10_f 10 4 -122328.4 -120615.1 3426.5

200_500_nopop_run_16_f 16 4 -122662.9 -120832.4 3661.0

200_500_nopop_run_17_f 17 5 -122080.9 -119913.1 4335.6

200_500_nopop_run_5_f 5 5 -122069.4 -119912.4 4314.0

200_500_nopop_run_11_f 11 5 -122002.1 -119869.4 4265.5

200_500_nopop_run_18_f 18 6 -122562.0 -119800.8 5522.4

200_500_nopop_run_6_f 6 6 -122802.4 -120200.2 5204.4

200_500_nopop_run_12_f 12 6 -122310.6 -119754.3 5112.5

Structure Harvester plots

Mean Ln (K)

Likelihood of observing the data under different values of K (number of genetic clusters). Y-axis shows the natural log likelihood (Figure 1). Lowest likelihood (closest to zero) indicates most likely number of genetic clusters in the data set.

Figure S1. Plot of mean L(K) against K

Evanno method ^2^

This approach examines the change in likelihood between successive K values (known as delta K) to improve estimates of the number of genetic clusters in the data set (Figure S2). This analysis provides very similar levels of support for K=3, K=4 and K=5 genetic clusters.

1. b)

Figure S2. a) Plot of the Rate of change of the mean likelihood of K against K. b) Plot of the Delta K against K.

References

1 Earl, D. A. & vonHoldt, B. M. STRUCTURE HARVESTER: a website and program for visualizing STRUCTURE output and implementing the Evanno method. *Conserv. Genet. Resour.* **4**, 359-361, doi:10.1007/s12686-011-9548-7 (2012).

2 Evanno, G., Regnaut, S. & Goudet, J. Detecting the number of clusters of individuals using the software structure: a simulation study. *Mol Ecol* **14**, 2611-2620, doi:<https://doi.org/10.1111/j.1365-294X.2005.02553.x> (2005).

Figure S3. Results of pairwise F_ST_ analysis of mitochondrial DNA control region haplotype data, across the six localities. Heat map shows increasing F_ST_ with blue shading. ‘X’ non-significant pairwise F_ST_ values, all other values significantly greater than zero (P<0.05).

Figure S4. Principal Coordinate Analysis results showing the distribution of nuclear data (SNPs) samples plotted along three axes (PC1 vs 2 – top; PC1 vs 3 – bottom).

Figure S5. Results of Bayescan analysis showing plot of pairwise F_ST_ values per SNP marker against probability of significant deviation from expected F_ST_ distribution for neutral loci. Markers shown to the right of the 5% and 1% probability lines display non-neutral patterns of differentiation.


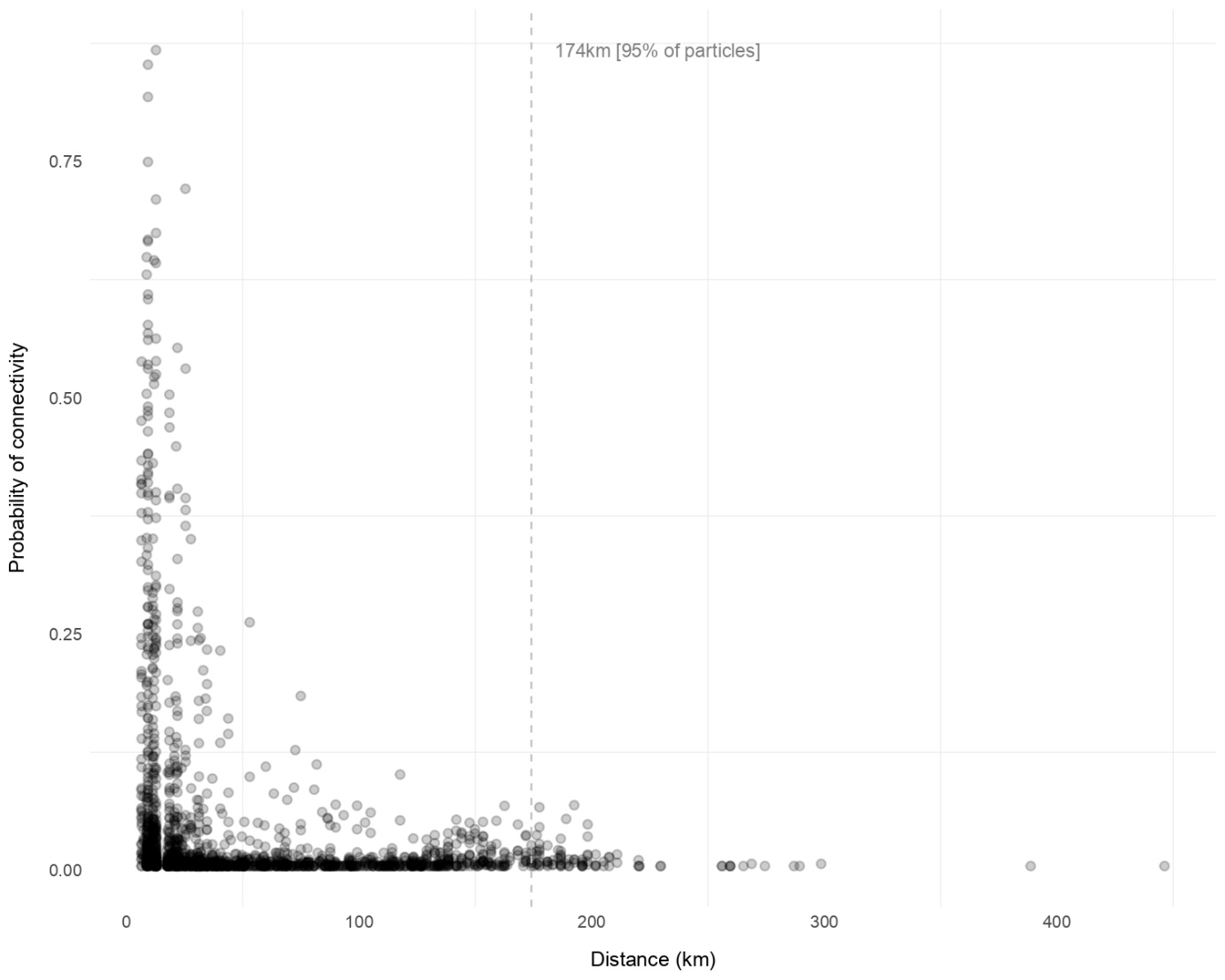


Figure S6. Probability of connectivity events of *Argyrosomus regius* larvae released from the known Atlantic spawning sites (Gironde, Tejo, Guadalquivir, Banc d’Arguin) with distance.

Table S3. Results of haplotype sequence diversity of control region mitochondrial DNA of *Argyrosomus regius* for the 86 samples across six localities. n=no. samples; H=no. haplotypes; Hr=rarefied no. haplotypes; Hd=haplotype diversity; uH=no. unique haplotypes; DHs=divergence from other populations

| **Locality** | **n** | **H** | **Hr** | **Hd** | **uH** | **DHs** |
| --- | --- | --- | --- | --- | --- | --- |
| Gironde | 15 | 4 | 2.00 | 0.37 | 0 | 0.57 |
| Tejo | 17 | 6 | 3.61 | 0.75 | 3 | 0.73 |
| Algarve | 14 | 5 | 3.63 | 0.81 | 1 | 0.75 |
| Guadalquivir | 16 | 4 | 2.24 | 0.58 | 0 | 0.66 |
| Banc d’Arguin | 14 | 9 | 5.99 | 0.90 | 4 | 0.79 |
| Senegal | 10 | 5 | 4.00 | 0.82 | 2 | 0.75 |
| **Total** | **86** | **16** | **4.99** | **0.81** | **-** | **-** |

Table S4. *Argyrosomus regius* pairwise (between locality) nuclear data F_ST_ values among the five sampling sites.  P-values <0.01 for all pairwise comparisons.

|  | **Gironde** | **Tejo** | **Algarve** | **Guadalquivir** | **Banc d’Arguin** |
| --- | --- | --- | --- | --- | --- |
| **Tejo** | 0.0192 |  |  |  |  |
| **Algarve** | 0.0289 | 0.0190 |  |  |  |
| **Guadalquivir** | 0.0296 | 0.0147 | 0.0046 |  |  |
| **Banc d’Arguin** | 0.0461 | 0.0366 | 0.0263 | 0.0270 |  |
| **Senegal** | 0.0690 | 0.0601 | 0.0461 | 0.0512 | 0.0271 |

Table S5. Sequence search results of the nine outlier loci identified through BayeScan analysis. BLASTn sequence searches returned hits against an existing set of genome-wide sequence data for the con-generic *Argyrosomus japonicus* for 8 of the 9 markers, but no close association with vertebrate protein databases (BLASTx).

| **Outlier no / SNP number** | **BLASTn result** | | | **BLASTx result** |
| --- | --- | --- | --- | --- |
|  | **Top species match** | **% sequence similarity** | **Coverage (bp)** |  |
| 1_0553 | *A. japonicus* | 100.00 | 63 | No significant match |
| 2_0128 | *A. japonicus* | 98.15 | 54 | No significant match |
| 3_0497 | *A. japonicus* | 100.00 | 69 | No significant match |
| 4_0909 | *A. japonicus* | 100.00 | 69 | No significant match |
| 5_0814 | *A. japonicus* | 100.00 | 69 | No significant match |
| 6_1417 | *A. japonicus* | 98.28 | 58 | No significant match |
| 7_0684 | *L. crocea* | 100.00 | 46 | No significant match |
| 8_0234 | *A. japonicus* | 93.10 | 58 | No significant match |
| 9_0593 | *A. japonicus* | 100.00 | 69 | No significant match |

Table S6. Genetic diversity statistics of *Argyrosomus regius* by sample locality, based on average values for 1534 SNP loci.  N=sample number; Na=number of alleles; Ne=effective number of alleles; Ho=observed heterozygosity; He=expected heterozygosity; PL=polymorphic loci.

| **Locality** | **N** | **Na** | **Ne** | **Ho** | **He** | **PL** |
| --- | --- | --- | --- | --- | --- | --- |
| Gironde | 12 | 1.83 | 1.46 | 0.32 | 0.28 | 88.33% |
| Tejo | 23 | 1.97 | 1.47 | 0.31 | 0.29 | 97.33% |
| Algarve | 12 | 1.94 | 1.54 | 0.43 | 0.32 | 94.00% |
| Guadalquivir | 16 | 1.95 | 1.45 | 0.29 | 0.28 | 94.46% |
| Banc d’Arguin | 17 | 1.91 | 1.45 | 0.28 | 0.27 | 91.07% |
| Senegal | 7 | 1.74 | 1.41 | 0.27 | 0.25 | 73.73% |

Table S7. Summary table of *Argyrosomus regius* tagged with biotelemetry transmitters. ^*^ Denotes pop-up satellite archival tags physically recovered. Fish #4 was recaptured at the tagging location 28 days after release. Two tags (#7 and #19) failed to transmit data.

| Fish # | TL (cm) | Tagging date | Programmed deployment (days) | Days attached | Sampling frequency (secs) | Distance between furthest locations | Tracking distance (km) |
| --- | --- | --- | --- | --- | --- | --- | --- |
| 1 | 131 | 20/09/2018 | 120 | 121 | 3 | 379 | 746 |
| 2 | 128 | 20/09/2018 | 180 | 181 | 5 | 135 | 536 |
| 3^*^ | 131 | 20/09/2018 | 180 | 166 | 5 | 381 | 576 |
| 4^*^ | 132 | 20/09/2018 | 180 | 28 | 5 | 51 | 97 |
| 5^*^ | 142 | 20/09/2018 | 120 | 110 | 3 | 281 | 662 |
| 6 | 127 | 20/09/2018 | 120 | 120 | 3 | 130 | 477 |
| 7 | 126 | 09/07/2019 | 300 | NA | 3 | - | - |
| 8^*^ | 122 | 09/07/2019 | 300 | 301 | 3 | 537 | 1857 |
| 17 | 143 | 27/09/2019 | 300 | 124 | 3 | 384 | 1298 |
| 18^*^ | 131 | 27/09/2019 | 300 | 83 | 3 | 135 | 318 |
| 19 | 112 | 27/09/2019 | 300 | NA | 3 | - | - |
| 20^*^ | 126 | 27/09/2019 | 300 | 300 | 3 | 419 | 2162 |
| 21 | 135 | 27/09/2019 | 300 | 300 | 3 | 155 | 1032 |
